# Sexual Satisfaction and Psychosocial Well-Being Among Saudi Survivors of Cervical and Breast Cancer: A Cross-Sectional Analysis

**DOI:** 10.3390/healthcare13192443

**Published:** 2025-09-26

**Authors:** Wedad M. Almutairi

**Affiliations:** Maternity and Child Department, Faculty of Nursing, King Abdulaziz University, Jeddah 21551, Saudi Arabia; walmutairi@kau.edu.sa

**Keywords:** cervical and breast cancer survivors, sexual function, psychosocial well-being, quality of life (QoL)

## Abstract

Background: While survival outcomes for breast and cervical cancer have improved in Saudi Arabia, little is known about the long-term sexual and psychosocial well-being of survivors. This study aimed to assess sexual satisfaction, emotional health, and social relationship quality among Saudi women diagnosed with cervical and breast cancers and to identify sociodemographic predictors of quality of life (QoL) across these domains. Methods: A cross-sectional survey was administered to 129 women with a history of breast or cervical cancer during May–July 2021. The instrument combined validated tools measuring three core QoL domains: sexual function and satisfaction, psychological and emotional well-being, and social and relationship qualities. Multivariable ordinal logistic regression was used. Results: A total of 129 women with cervical and breast cancers (51.2% cervical, 48.8% breast) participated. Most were aged 31–45 years (45.7%), married (83.0%), with 48.1% holding a bachelor’s degree. Overall, 74.4% of participants reported high to moderate emotional well-being; 48.8% reported satisfactory sexual function, and only 41.1% perceived high quality in social relationships. Younger age (21–30 years), higher education, and having more children were significantly associated with lower emotional well-being (*p* < 0.05). Conversely, current treatment status and higher parity were associated with better sexual function. Social and relationship quality was significantly higher among younger and employed women. Perceived importance of domains varied, with emotional well-being rating the highest (82.9%). Conclusions: Sexual and social QoL are underserved yet critical components of survivorship care. Cultural, familial, and educational contexts play significant roles in shaping post-treatment experiences. Interventions tailored to age, family dynamics, and treatment stage are needed to improve the holistic well-being of survivors in Saudi Arabia.

## 1. Introduction

Breast and cervical cancers are among the major health concerns for women worldwide. While cervical cancer is among the top 10 cancers affecting Saudi women, breast cancer is the most diagnosed cancer among women in Saudi Arabia, accounting for almost 30% of all cancer cases [1,2]. Significant disparities still exist in addressing the long-term quality of life (QoL) of survivors, particularly in the areas of sexual health and interpersonal relationships, despite growing awareness and developments in early detection and treatment.

Sexual health is a fundamental aspect of QoL but remains under-explored and often stigmatized, specifically in conservative societies. Treatments for breast and cervical cancers such as mastectomy, chemotherapy, pelvic radiation, and hormone therapy are well known to induce adverse sexual consequences, including reduced libido, vaginal dryness, dyspareunia, anorgasmia, and body image issues [3,4]. These problems not only compromise physical but also emotional connections with partners, therefore possibly causing distress and marital strife. A recent study in Saudi Arabia highlighted that although very few sought professional advice owing to sociocultural constraints, a large percentage of the participant women indicated notable changes in sexual desire and satisfaction following treatment for cervical and breast cancers [5].

In addition to sexual dysfunction, psychological distress is a prevalent and persistent consequence of cervical and breast cancer survivorship. Feelings of fear, sadness, anxiety, and hopelessness are common across the cancer continuum, often exacerbated by fears of recurrence and uncertainty about the future [6]. Importantly, psychological symptoms can significantly worsen sexual dysfunction and reduce treatment adherence, yet access to professional psychosocial support remains limited in many healthcare settings. In Saudi Arabia, where public discussion of mental health is still evolving, cultural stigma and lack of awareness pose additional barriers to seeking support [7,8].

Social and relational well-being is another key, yet often overlooked, pillar of survivorship. Cancer diagnosis and treatment frequently disrupt women’s roles within the family, workforce, and community, contributing to isolation, diminished self-worth, and strained relationships [9]. Evidence suggests that strong social support networks serve as a buffer against psychological distress and improve treatment outcomes. However, in patriarchal societies, women may hesitate to express vulnerability or seek emotional support, particularly regarding sexual or intimate concerns, for fear of social judgment or familial pressure.

Saudi Arabia presents a unique cultural landscape where the intersection of gender norms, family structure, religion, and privacy strongly shapes women’s healthcare experiences [10]. While the population is nearly evenly split by gender, women’s health needs, particularly in areas such as sexuality, are often underexplored [10]. Notably, the survival rate for breast and cervical cancers has increased substantially in recent years, driven by improved awareness, screening programs, and access to treatment [11,12]. However, despite this positive trend, studies addressing the emotional and sexual health of female cancer survivors remain sparse. Moreover, sexual health is often under-discussed in clinical encounters, reflecting broader cultural hesitancy to address intimate health topics within conservative societies [13].

Therefore, this study aims to assess the quality of sexual life, psychological well-being, and social relationship satisfaction among Saudi women diagnosed with breast or cervical cancers. Using culturally contextualized and validated instruments, this research will contribute much-needed insights into the post-treatment experiences of cervical and breast cancer survivors in Saudi Arabia and inform future interventions designed to improve QoL in this medically and socially vulnerable population.

## 2. Materials and Methods

Study Design and Setting. This was a cross-sectional, survey-based study designed to assess sexual function and satisfaction, psychological well-being, and social relationship quality among women previously diagnosed with cervical and breast cancers in the Kingdom of Saudi Arabia. Data collection occurred between May and July 2021. This study adhered to the STROBE (Strengthening the Reporting of Observational Studies in Epidemiology) guidelines for transparent reporting of observational studies [14]. Participants and Eligibility Criteria are as follows:

Eligible participants were (1) women aged ≥ 18 years, (2) with a confirmed prior diagnosis of breast or cervical cancer, (3) having completed primary cancer treatment (e.g., surgery, chemotherapy, radiation) or ongoing treatment, and (4) fluent in Arabic. Women with active cancer recurrence were excluded. We utilized social media platforms to disseminate the survey invitation to individuals who met the eligibility criteria for our study. Participants who responded affirmatively to the initial screening question were subsequently contacted and invited to complete the full survey. Data was collected anonymously and manually entered into a secure digital database on the researcher’s computer.

Survey Development and Instrumentation: This survey was developed by adapting and combining several validated instruments:

- The Satisfaction Survey, designed to assess sexual satisfaction and function in gender-diverse populations post-vaginoplasty, contributed foundational domains on genital satisfaction, desire, and ejaculation [15,16].
- The Quality of Sexual Life Questionnaire for breast cancer survivors in Mainland China provided items on orgasmic intensity, partner satisfaction, and sexual normalcy, ensuring cross-domain comparability [4].

The final instrument included four core domains:

1. Sexual Function and Satisfaction (8 items);
2. Psychological and Emotional Well-Being (3 items);
3. Social and Relationship Quality (3 items);
4. Importance of Each Domain (14 items rated separately).

Items were rated on a 5-point Likert scale (1 = very poor to 5 = excellent), with higher scores reflecting better perceived quality in that domain. An example of a 5-point Likert scale question: “Concerning how you feel before starting a sexual activity, how do you think things are going”? To enrich the interpretation of domain scores and incorporate a patient-centered lens, we added a novel section evaluating the perceived importance of each domain. Participants were asked to rate, on a 4-point Likert scale (1 = not important, 2 = slightly important, 3 = important, 4 = extremely important), how much each domain contributed to their quality of life. An example of a 4-point Likert scale question: “In your life, what do you consider this to be”? This component was not part of the original reference instruments [4,14,15] but was included to allow for a better contextualization of domain-specific satisfaction and to identify priority areas for survivorship care. This addition is consistent with emerging recommendations in patient-reported outcome (PRO) methodology, which emphasize the importance of assessing both experience and value attribution when measuring quality of life in oncology settings. Capturing what participants consider most important enables a more nuanced, patient-driven interpretation of QoL gaps—particularly in populations with diverse cultural and personal needs [17].

### 2.1. Translation, Content Validation, and Reliability

To ensure linguistic and cultural validity, this survey was translated from English to Arabic using forward and backward translation, based on World Health Organization (WHO) recommendations for tool translation [18]. The translation was reviewed by bilingual researchers and a certified professional translator. A pilot study was then conducted with 20 Saudi women to assess content clarity, cultural acceptability, and face validity. Based on participant feedback and expert review by oncology and public health specialists, minor modifications were made to item phrasing to improve readability and cultural sensitivity. This process enhanced both content and construct validity, ensuring alignment with the target population [19,20].

Sample size estimation was guided by methodological standards for cross-sectional studies involving multivariable ordinal regression. A minimum of 10–15 participants per predictor variable was used as a rule of thumb to ensure stable estimates in multivariable modeling [21]. Considering 8 to 10 key independent variables of interest, such as age, marital status, education level, number of children, and cancer type, the target sample size was calculated to be at least 100 to 120 participants. This range was deemed adequate to detect medium effect sizes with sufficient power (≥80%) and maintain model reliability while accounting for potential nonresponse and missing data. Ultimately, 129 women participated in this study, exceeding the minimum required sample size. The response rate was 90%, reflecting high participant engagement and the effectiveness of the in-person recruitment strategy implemented during outpatient follow-up visits at the oncology center. A convenience sampling approach was employed, wherein eligible participants were recruited through an online survey.

Data Collection Participants completed the survey anonymously online through a secure web-based platform. Informed electronic consent was obtained from all respondents prior to participation.

Ethical Considerations: The study protocol was reviewed and approved by the Nursing Research Ethics Committee at the Faculty of Nursing at King Abdulaziz University, under approval number Ref No 1F.21.

### 2.2. Variables and Measurement

- Primary outcomes were total scores in each of the three domains (sexual function, psychological well-being, and social relationships);
- Domain scores were also dichotomized for descriptive purposes: scores ≥ 3 were classified as “High Satisfaction” and <3 as “Moderate/Low Satisfaction [4,14,15];
- Independent variables included age, marital status, education level, employment status, cancer type (breast or cervical), age at diagnosis, time since diagnosis, and treatment received.

Statistical Analysis: Descriptive statistics were used to summarize the sociodemographic and clinical characteristics of the study population. Categorical variables were reported as frequencies and percentages. The aim was to describe the population’s distribution across key characteristics, including age at diagnosis, time since diagnosis, number of children, and cancer type. To assess factors associated with quality of sexual life among women with cervical and breast cancers, we constructed three separate multivariable ordinal logistic regression models—one for each domain: (1) Sexual Function and Satisfaction; (2) Psychological and Emotional Well-Being; and (3) Social and Relationship Qualities. Each outcome was based on composite Likert-scale scores categorized into ordinal levels. Independent variables included age group, marital status, educational attainment, employment status, cancer type, treatment status, time since diagnosis, and number of children.

We evaluated the assumptions of ordinal logistic regression, including the proportional odds (parallel lines) assumption, using the Brant test. No significant violations were detected. Multicollinearity among predictors was assessed using the Variance Inflation Factor (VIF), and all values were below the conservative threshold of 5, indicating acceptable independence of predictors. Given the relatively small number of observations in some categorical subgroups (e.g., graduate education level, certain age/treatment combinations), we applied variable collapsing and category merging strategies to reduce sparse cell counts and improve model stability. Specifically, categories with <10% of total observations were either merged with adjacent groups or retained only if they were clinically meaningful. This approach preserved statistical power while maintaining interpretability. Where appropriate, Firth’s penalized maximum likelihood estimation was considered as a sensitivity check to reduce small-sample bias in maximum likelihood estimates [22]. Pseudo R-squared ranged between 0.6 and 0.65, which represents good explanatory power. AIC was 327.37, which is considered good compared to the model without variable collapsing, AIC 522.1. Regression results were reported as odds ratios (ORs) with 95% confidence intervals (CIs). Statistical significance was defined as a 2-sided *p*-value < 0.05. Analyses were conducted using R software (v4.2.3) and SAS (9.4).

Data were reviewed for completeness prior to analysis. Participants with missing responses on key variables were excluded using the listwise deletion method. The proportion of missing data was minimal and did not impact the overall sample size or statistical power.

## 3. Results

### 3.1. Descriptive Characteristics of the Study Population

A total of 129 women diagnosed with cervical and breast cancers (51.2% cervical, 48.8% breast) participated in this study. Most participants were between the ages of 31 and 45 years (45.7%), followed by those aged 21 to 30 years (33.3%), and those older than 45 years (20.9%). Most participants were married (83.0%), held a bachelor’s degree (48.1%), and were Saudi nationals (89.9%). Regarding occupational status, 56.6% were non-employed or retired. In terms of family structure, 59 (45.7%) reported having 1 to 3 children; 50 (38.8%) had 4 to 10 children, and 20 (15.5%) had no children. Nearly half the women (46.5%) were diagnosed with cancer between the ages of 21 and 30 years, and 61% were first diagnosed between 1 and 3 years (Table 1).

To assess the internal consistency of the final Arabic version of the instrument, Cronbach’s alpha was calculated for the overall scale following pilot testing. The instrument demonstrated excellent reliability, with a Cronbach’s alpha coefficient of 0.89, exceeding the commonly accepted threshold of 0.70 for group-level comparisons [19]. This supports the internal coherence of the multi-item domains and justifies the use of composite domain scores in further analysis.

### 3.2. Perceived Quality of Sexual Life Across the Three Domains

**Figure 1:**
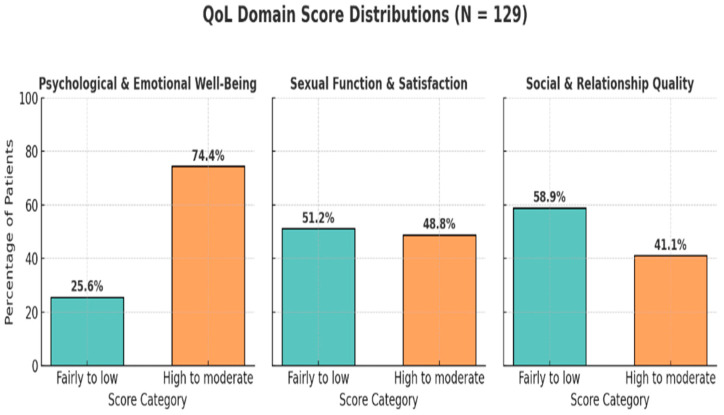
Quality of Sexual Life Questionnaire domain scores.

When assessing the perceived quality of sexual life across the three measured domains, most participants (74.4%) reported high-to-moderate psychological and emotional well-being, indicating generally favorable mental health among the cohort. In contrast, less than half (48.8%) of women reported high-to-moderate levels of sexual function and satisfaction, highlighting a significant burden of sexual dysfunction post-treatment. The domain with the lowest perceived quality was social and relationship qualities, with only 41.1% rating it as high-to-moderate, while a notable 58.9% described their social connectedness and interpersonal relationships as low, underscoring potential challenges in post-treatment social reintegration and support (Figure 1).

### 3.3. Perceived Importance of QoL Domains

**Figure 2:**
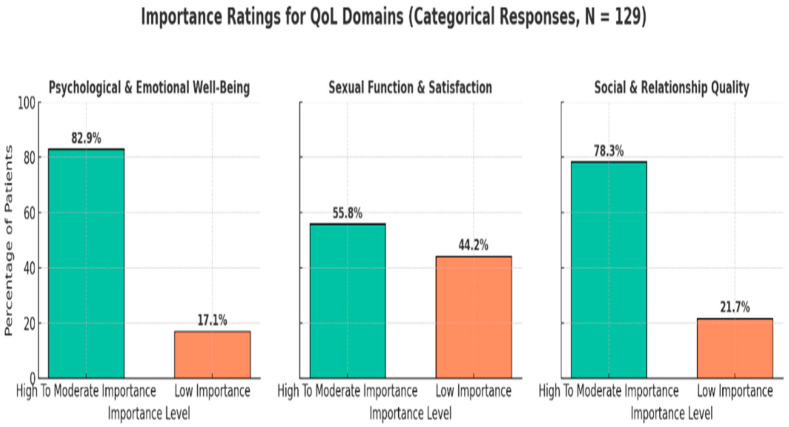
Importance score for each outcome.

Most participants perceived psychological and emotional well-being to be of high or moderate importance (82.9%), with only 17.1% rating it as of low importance. Similarly, 78.3% considered social and relationship qualities to be of high-to-moderate importance, while 21.7% rated them as less important. In contrast, sexual function and satisfaction showed a more divided perception; 55.8% rated them as highly important, while 44.2% viewed them as of low importance. (Figure 2).

### 3.4. Bivariate Analysis for the Three Domains Across Sociodemographic Variables

**Figure 3:**
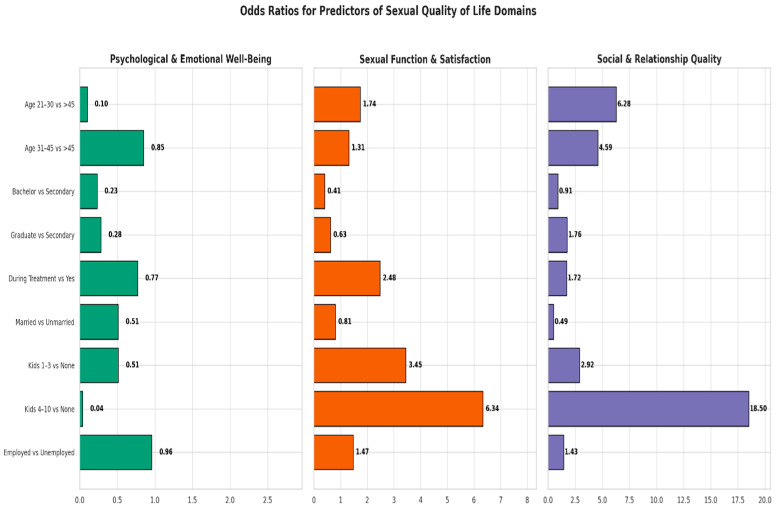
Multivariable Ordinal Logistic Regression Results for Predictors of Sexual Quality of Life Domains Among Women Diagnosed with Breast or Cervical Cancer in Saudi Arabia.

Chi-square analysis revealed several significant associations between sociodemographic variables and domains of quality of sexual life among cervical and breast cancer survivors. In the domain of Psychological and Emotional Well-Being, the number of children was significantly associated with well-being levels (*p* = 0.024). Specifically, 82.4% of women with no children reported high-to-moderate psychological well-being, compared to only 60.7% among those with four to ten children. In the domain of Sexual Function and Satisfaction, educational level was significantly associated with satisfaction scores (*p* = 0.043). A higher proportion of women with a bachelor’s degree (55.8%) reported high-to-moderate sexual satisfaction compared to only 38.9% among those with high school education or less. Finally, in the domain of Social and Relationship Quality, both marital and occupational status were significantly associated with perceived relationship quality (*p* = 0.011 and *p* = 0.039, respectively). Married women reported higher levels of social and relationship quality (45.2% rated high to moderate) compared to 22.2% among divorced or widowed participants. Likewise, 50.0% of employed women reported high-to-moderate relationship quality compared to 36.1% among the unemployed. These findings highlight the influence of key sociodemographic factors on different dimensions of psychosocial and sexual well-being in this population (Figure 3).

### 3.5. Predictors of Sexual Quality of Life (Multivariate Analysis)

Across the three domains, Psychological and Emotional Well-Being, Sexual Function and Satisfaction, and Social and Relationship Qualities, several variables emerged as significant predictors of sexual quality of life among Saudi women diagnosed with breast or cervical cancer. Notably, age, educational level, and treatment status were significant in multiple models. Women aged 31–45 years consistently reported either improved or reduced outcomes depending on the domain, highlighting the complexity of survivorship at midlife. Additionally, marital status and number of children were uniquely associated with relational and sexual outcomes, reflecting culturally shaped experiences of intimacy and support (Table 2 and Figure 3).

### 3.6. Psychological and Emotional Well-Being

Women aged 21–30 years had significantly lower odds of reporting better psychological and emotional well-being scores compared to those older than 45 years (OR, 0.1; 95% CI, 0.01–0.58; *p* = 0.01). Similarly, those with a bachelor’s degree were less likely to report favorable psychological outcomes compared to women with a secondary education or less (OR, 0.23; 95% CI, 0.06–0.84; *p* = 0.02). Having more than four children was also associated with decreased odds of reporting high psychological well-being (OR, 0.038; 95% CI, 0.004–0.35; *p* = 0.004) (Table 2, Figure 3).

### 3.7. Sexual Function and Satisfaction

Those with a bachelor’s degree were less likely to report favorable psychological outcomes compared to women with a secondary education or less (OR, 0.41; 95% CI, 0.16–0.98; *p* = 0.04). Women currently receiving treatment had significantly greater odds of reporting better sexual outcomes compared to those who had completed treatment (OR, 2.48; 95% CI, 1.11–5.56; *p* = 0.02). Having 4 to 10 children also positively influenced sexual satisfaction (OR, 6.35; 95% CI, 1.33–30.25; *p* = 0.02) (Table 2, Figure 3).

### 3.8. Social and Relationship Quality

Women aged 21–30 years, (OR, 6.29; 95% CI, 1.38–28.73; *p* = 0.03) and those 31 to 45 years old (OR, 4.59; 95% CI, 1.18–17.93; *p* = 0.02) also reported significantly better outcomes compared to those older than 45 years. Having 4 to 10 children also positively influenced Social and Relationship Qualities positively, (OR, 18.5; 95% CI, 3.42–100.85; *p* = 0.0007); those with more than four children were more likely to report better social relation scores compared to those who had no children (Figure 3).

## 4. Discussion

This study offers critical insight into the multifaceted dimensions of sexual and psychosocial quality of life among Saudi women diagnosed with cervical and breast cancers, specifically breast and cervical cancers. By examining sexual function, emotional well-being, and social relationships through validated, culturally contextualized measures, we identified key sociodemographic and clinical predictors shaping these domains. These findings contribute to the expanding literature that emphasizes the need to move beyond survival metrics and toward patient-centered survivorship care in oncology. In this study of a sample of Saudi women diagnosed with breast or cervical cancer, participants reported variable satisfaction across the three core domains of sexual quality of life. Psychological and emotional well-being had the highest reported satisfaction (74.4%), followed by sexual function and satisfaction (48.8%) and social and relationship qualities (41.1%). These findings are consistent with prior studies conducted in both regional and global settings, which have shown that while emotional resilience often remains relatively intact, sexual and relational dimensions of quality of life tend to suffer more post-treatment [23,24,25].

### 4.1. Predictors Across Domains

Age, education level, and number of children emerged as consistent predictors across multiple domains, highlighting their integral roles in shaping post-treatment quality of life. Women aged 31 to 45 years exhibited variable outcomes depending on the domain, while those younger than 30 years reported particularly poor psychological well-being. These findings align with earlier research suggesting that younger women face heightened emotional vulnerability following a cancer diagnosis due to concerns about body image, fertility, and disrupted life expectations [26]. Similarly, higher educational attainment, specifically holding a bachelor’s degree, was paradoxically associated with lower odds of favorable outcomes across psychological and sexual domains.

### 4.2. Psychological and Emotional Well-Being

Nearly three-quarters (74.4%) of participants rated their emotional well-being as high-to-moderate. However, regression analysis showed that women aged 21–30 years were significantly less likely to report favorable outcomes compared to those older than 45 years. This result repeats the global literature, indicating that younger cancer survivors are more susceptible to emotional distress, potentially due to perceived loss of femininity, altered life trajectories, or unfulfilled social roles [27,28,29]. Notably, women with bachelor’s degrees were also less likely to report emotional well-being, suggesting a potential mismatch between expectations and support. In Saudi Arabia, where mental health remains under-discussed, more educated women may struggle silently with the emotional toll of illness, feeling pressure to maintain composure within social and familial structures [29,30]. Additionally, the number of children was associated with emotional outcomes. Women with four or more children reported significantly lower psychological well-being than those with none. This may be attributed to increased caregiving burdens or challenges in balancing health recovery with domestic responsibilities, particularly in patriarchal societies where women are primary caregivers [31].

### 4.3. Sexual Function and Satisfaction

Sexual function was the domain with the lowest psychological well-being reported satisfaction, with only 48.8% indicating high or moderate satisfaction. Treatment status, education level, and number of children were significant predictors. Women currently undergoing treatment reported higher odds of sexual satisfaction than those who had completed treatment. While initially counterintuitive, this may reflect increased clinical contact and emotional support during active treatment, compared with the lack of structured follow-up post-treatment, a pattern previously noted in global oncology care [25]. Emerging evidence highlights the physiological impact of stress on sexual health, with studies showing reduced heart rate variability and psychological maladjustment among cancer survivors [32]. These findings support our results, where diminished emotional well-being was significantly associated with lower sexual satisfaction among gynecologic cancer survivors. Women with 4 to 10 children also had significantly higher odds of reporting sexual satisfaction. In Saudi culture, motherhood is often linked to social validation and marital stability, which may enhance feelings of sexual normalcy and fulfillment [25]. Conversely, women with a bachelor’s degree were significantly less likely to report high satisfaction, possibly reflecting greater awareness of dysfunction, unmet needs, or cultural expectations of intimacy that remain unaddressed in healthcare encounters [25].

### 4.4. Social and Relationship Quality

Social and relational well-being had the lowest satisfaction rates, with only 41.1% of participants reporting favorable outcomes. Yet, younger and middle-aged women (21–45 years) reported significantly better social outcomes than those older than 45. This could be attributed to stronger social networks, greater family integration, or more active roles in their communities. In Saudi Arabia, familial structures, especially among younger women, can offer significant emotional buffering despite the presence of illness, potentially explaining the higher social scores [25]. Moreover, women with 4 to 10 children had markedly higher odds of favorable relational quality. As previous studies have shown, motherhood enhances familial connectedness, serves as a source of identity and pride, and may provide a sense of relational purpose post-treatment [25]. However, the directionality of this relationship warrants further qualitative exploration, particularly regarding whether perceived social support is a cause or consequence of high relational satisfaction.

The experiences of Saudi breast and cervical cancer survivors concerning sexual satisfaction and psychological well-being might also be influenced by cultural and religious elements, in addition to disparities in healthcare systems. Cultural traditions in Saudi Arabia frequently restrict open discussion regarding sexuality, while religious convictions significantly shape views on sexual duties and modesty, potentially impacting women’s perceptions and reports of sexual satisfaction. Moreover, cultural expectations related to femininity, fertility, and marital obligations may worsen women’s emotions after a diagnosis of cervical and breast cancers. The stigma surrounding sexual health discussions, along with restricted access to culturally sensitive counseling services, clearly sets the Saudi environment apart from other nations and highlights the necessity for localized, culturally suitable support measures.

### 4.5. Limitations

This study is subject to several limitations. First, its cross-sectional design precludes the establishment of causal relationships between predictors and sexual quality of life outcomes. Second, the sample size was relatively small and recruited from a single tertiary care center using convenience sampling, which may limit the generalizability of the findings to the broader population of cervical and breast cancer survivors in Saudi Arabia. Finally, one limitation of this study is the reliance on self-reported survey data with convenience sampling, which may be subject to recall bias, selection bias, or social desirability bias, potentially affecting the accuracy of responses.

## 5. Conclusions

Overall, this study underscores the complex interplay between sociodemographic factors and quality-of-life domains among Saudi cervical and breast cancer survivors. While emotional well-being was generally rated favorably, sexual satisfaction and relational outcomes revealed significant gaps, particularly among younger, more educated women. These findings emphasize the need for culturally competent survivorship models in Saudi Arabia that include structured sexual counseling, psychosocial support, and family-oriented interventions as a clinical implication of our findings. Future research should investigate longitudinal trajectories and explore targeted interventions that reflect the unique cultural and societal expectations of women in the region.

## Figures and Tables

**Table 1:** Descriptive Characteristics of the Study Population.

Variable	Category	N (%) ^1^
Age	21 to 30 Years	43 (33.33)
	31 to 45 Years	59 (45.74)
	More than 45 Years	21 (20.93)
Marital Status	Married	107 (82.95)
	Non-Married	22 (11.05)
Educational Level	Bachelor	62 (48.06)
	Graduate studies	13 (10.08)
	Secondary or Less	54 (41.86)
Nationality	Saudi	116 (89.92)
	Non-Saudi	13 (10.08)
Occupational Status	Employed	56 (43.41)
	Non-employed or retired	73 (56.59)
Number of children	From 1 to 3	59 (45.74)
	From 4 to 10	50 (38.76)
	None	20 (15.5)
Age at your first diagnosis	From 21 to 30 Years	60 (46.51)
	From 31 to 45 Years	48 (37.21)
	More than 45 Years	21 (16.28)
When were you diagnosed with cervical or breast cancer?	From 1 to 3 years	79 (61.24)
	More than 3 years	26 (20.16)
	Less than a year	23 (17.83)
Type of diagnosed cancer		
	Breast cancer	63 (48.84)
	Cervical cancer	66 (51.16)
Sexuality score		
Psychological and Emotional Well-Being	Fair-to-low	33 (25.58)
	Hig-to-moderate	96 (74.42)
Sexual Function and Satisfaction	Fair-to-low	66 (51.16)
	High-to-moderate	63 (48.84)
Social and Relationship Qualities	Fair-to-low	76 (58.91)
	High-to-moderate	53 (41.09)

^1^ N (%) = sample size and percentage.

**Table 2:** Multivariable Ordinal Logistic Regression Results for Predictors of Sexual Quality of Life Domains Among Women Diagnosed with Breast or Cervical Cancer in Saudi Arabia.

Variable	Psychological and Emotional Well-Being			Sexual Function and Satisfaction			Social and Relationship Qualities		
	OR ^1^	95% CI ^2^	*p*-Value	OR	95% CI	*p*-Value	OR	95% CI	*p*-Value
Age: From 21 to 30 Years vs. More than 45 Years	0.1	(0.01, 0.58)	0.01	1.74	(0.46, 6.61)	0.41	6.28	(1.37, 28.72)	0.01
Age: From 31 to 45 Years vs. More than 45 Years	0.85	(0.17, 4.07)	n.s	1.31	(0.41, 4.25)	0.64	4.59	(1.17, 17.93)	0.02
Educational Level: Bachelor vs. Secondary or Less	0.23	(0.06, 0.84)	0.02	0.41	(0.16, 0.98)	0.04	0.91	(0.36, 2.26)	n.s
Educational Level: Graduate studies vs. Secondary or Less	0.28	(0.03, 2.06)	n.s	0.63	(0.14, 2.8)	0.54	1.76	(0.36, 8.61)	n.s
Have you been treated/ am now/under treatment vs. Yes	0.77	(0.23, 2.48)	n.s	2.48	(1.11, 5.56)	0.02	1.72	(0.76, 3.92)	n.s
Marital Status: Married vs. Unmarried	0.51	(0.09, 2.88)	n.s	0.81	(0.28, 2.32)	0.69	0.49	(0.16, 1.51)	n.s
Number of children: From 1 to 3 vs. None	0.513	(0.08, 3.15)	n.s	3.45	(0.93, 12.75)	0.06	2.92	(0.86, 9.86)	n.s
Number of children: From 4 to 10 vs. None	0.038	(0.004, 0.35)	0.00	6.34	(1.33, 30.24)	0.02	18.5	(3.42, 100.85)	0.00
Occupational Status: Employed vs. Unemployed	0.959	(0.29, 3.17)	n.s	1.47	(0.61, 3.53)	0.38	1.43	(0.58, 3.51)	n.s

Age, education, treatment status, and number of children are the significant predictors overlapping the three domains. ^1^ Odds ratio (OR); ^2^ 95% Confidence interval (95% CI); *p*-values; n.s = not significant.

## Data Availability

All data available in the atrical.
